# Single Cell Synchrotron FT-IR Microspectroscopy Reveals a Link between Neutral Lipid and Storage Carbohydrate Fluxes in *S. cerevisiae*

**DOI:** 10.1371/journal.pone.0074421

**Published:** 2013-09-11

**Authors:** Frédéric Jamme, Jean-David Vindigni, Valérie Méchin, Tamazight Cherifi, Thierry Chardot, Marine Froissard

**Affiliations:** 1 INRA, UAR 1008, CEPIA, Nantes, France; 2 Synchrotron SOLEIL, Gif-sur-yvette, France; 3 INRA, UMR1318, Institut Jean-Pierre Bourgin, Versailles, France; 4 AgroParisTech, UMR1318, Institut Jean-Pierre Bourgin, Versailles, France; Fundação Oswaldo Cruz, Brazil

## Abstract

In most organisms, storage lipids are packaged into specialized structures called lipid droplets. These contain a core of neutral lipids surrounded by a monolayer of phospholipids, and various proteins which vary depending on the species. Hydrophobic structural proteins stabilize the interface between the lipid core and aqueous cellular environment (perilipin family of proteins, apolipoproteins, oleosins). We developed a genetic approach using heterologous expression in *Saccharomyces cerevisiae* of the *Arabidopsis thaliana* lipid droplet oleosin and caleosin proteins AtOle1 and AtClo1. These transformed yeasts overaccumulate lipid droplets, leading to a specific increase in storage lipids. The phenotype of these cells was explored using synchrotron FT-IR microspectroscopy to investigate the dynamics of lipid storage and cellular carbon fluxes reflected as changes in spectral fingerprints. Multivariate statistical analysis of the data showed a clear effect on storage carbohydrates and more specifically, a decrease in glycogen in our modified strains. These observations were confirmed by biochemical quantification of the storage carbohydrates glycogen and trehalose. Our results demonstrate that neutral lipid and storage carbohydrate fluxes are tightly connected and co-regulated.

## Introduction

In yeasts, plants and other organisms, storage lipids, i.e. oil, are packaged into specialized structures called lipid droplets or oil bodies [1,2]. These consist mainly of a core of neutral lipids (triacylglycerols and/or steryl esters) surrounded by a monolayer of phospholipids, and contain a number of proteins which vary considerably with the species [3,4]. Proteomic and genetic studies of this compartment in the last decade have shown that lipid droplets are not inert fat depots. Instead, they appear as a complex dynamic organelle with a role in metabolism control and cell signaling [5–7]. These observations suggest that lipid droplet proteins could be worthwhile targets in biotechnological approaches to modify neutral lipid dynamics in cells. An understanding of the mechanisms governing lipid droplet morphology or neutral lipid storage in improved biological models would be helpful for development in this area. Data collected in the yeast model *S. cerevisiae* is relevant to the SCO (single cell oil) technologies in oleaginous microorganisms such as yeasts or algae [8,9].

Recent studies in *S. cerevisiae* showed that lipid droplets are highly plastic and various mutant strains show a pronounced increase in neutral lipid storage [10,11]. In our laboratory, we also observed neutral lipid accumulation using heterologous expression of an *Arabidopsis thaliana* lipid droplet protein. The most abundant *A. thaliana* seed lipid droplet proteins are small alkaline proteins (15-21 kDa) called oleosins. Oleosins are characterized by a conserved hydrophobic central domain of 70 residues, the longest found in known proteins, flanked by hydrophilic N and C termini of variable sizes [12,13]. All experimental and computational data agree with modeling oleosins as interfacial proteins with their termini at the lipid droplet surface and a hydrophobic central region spanning the monolayer and probably digging into the neutral lipid core [14–20]. This original structure suggested that oleosins may be implicated in lipid droplet biogenesis and stabilization. It has been shown that oleosins effectively control oil body size and lipid accumulation *in planta* [21,22]. Interestingly, caleosin, one of the minor proteins of seed lipid droplets, displays similar sequence organization with oleosin [23,24]. It shares, as the oleosin family, capacity to stabilize artificial lipid droplets [25] and *in vivo*, it was demonstrated that during germination it plays a role in degradation of lipid storage [26]. Yeast was successfully used to decipher some of the oleosin properties and functions [27–30]. Authors did not reveal any potential effect on lipid droplet morphogenesis but Parthibane et al. observed an increase in neutral lipids when a peanut oleosin isoform was expressed in yeast [31]. We also demonstrated that the expression of a GFP-tagged version of the caleosin AtClo1 induced lipid droplet proliferation and an increase in total fatty acid content due to an overaccumulation of triacylglycerols and steryl esters [32].

In yeasts and other organisms, pathways leading to the synthesis and degradation of neutral lipids, from acetyl-CoA to lipid esters and reverses, are now well known [33,34]. The biochemistry of lipid accumulation has been extensively investigated in oleaginous yeasts. Under nutrient-limiting conditions with high carbon, oil accumulation is enhanced, with the most significant increases under nitrogen limitation. Some key enzymes were also identified [35,36]. Nevertheless, the underlying controls and signaling cascades which regulate neutral lipid pools remain to be clearly elucidated.

To gain a clearer picture of the mechanisms involved in neutral lipid storage, a general view of metabolism throughout neutral lipid accumulation in cells with contrasting oil content is needed. The first attempts were proteomics of lipid droplets isolated from cells with varying oil content [37–39]. Recently, this approach was combined with lipidomic analyses [40] to reveal that the combination of lipids and proteins associated with this organelle is highly dynamic. In 2011, transcriptomics of the oleaginous yeast, *Y. lipolytica*, during the transition from biomass production to lipid accumulation was also carried out [41]. The authors concluded that lipid accumulation is concomitant with a repression of protein production and a rerouting of carbon fluxes, rather than increased lipid metabolism. Cell metabolism and composition can also be explored using synchrotron FT-IR (Fourier transformed-infrared) microspectroscopy. FT-IR is a non-invasive technique for monitoring biochemical changes *in situ* in cells and tissues. This powerful analytical technique gives spectral fingerprints of biological macromolecules such as lipids, nucleic acids, and carbohydrates, and is therefore sensitive to structural and compositional changes in tissues [42–44]. FT-IR appears as an emerging technique for cell lipid content monitoring and cell screening. For example, in the context of SCO technology (biofuel and green chemistry), FT-IR was developed to replace traditional lipid analyses which require large amounts of biomass, are solvent-consuming, and are not particularly effective for the analysis of a large number of samples [45–48]. In addition, the high spectral and spatial resolution offered by synchrotron infrared radiation allowed analysis at the single cell level (3-5 microns). Thus, heterogeneity of cells population can be studied and clearly represented by multivariate analysis as shown in score plot figures. This approach was successfully used to follow the biochemical changes induced by nanosilver treatment of single *S. cerevisiae* [49] offering access to multivariate statistical analysis.

In the present study, we exploited *S. cerevisiae* strains with enhanced neutral lipid contents and investigated their biochemical composition at the single cell level using synchrotron FT-IR microspectroscopy. In strains expressing GFP-tagged versions of the *A. thaliana* lipid droplet proteins oleosin (AtOle1) and caleosin (AtClo1), we observed different phenotypes for lipid droplet morphology and triacylglycerol and steryl ester accumulation. We obtained an overview of the metabolism changes induced by lipid overaccumulation using single cell FT-IR microspectroscopy. Multivariate statistical analysis of the spectra showed a clear effect on carbohydrate pools and specifically reduced glycogen in our modified strains. These observations were confirmed using biochemical quantification of the storage carbohydrates glycogen and trehalose.

## Materials and Methods

### Yeast strains and growth conditions

The yeast strain used throughout this study was BY 4741 (Mata, *his3Δ*, *leu2Δ*, *met15Δ*, *ura3Δ*) from Euroscarf. This strain was transformed with either pGAL-GFP (alias pNBT29, *URA3*, multicopy) [50], pGAL-OLE1-GFP (this study) or pGAL-CLO1-GFP [32] using the LiAc/Salmon sperm Carrier DNA/ PEG method described by Gietz et al. [51]. Cells were grown in minimal medium (YNB) containing 0.67% yeast nitrogen base without amino acids and ammonium sulfate, supplemented with 5 g.L^-1^ ammonium sulfate and 0.2% casaminoacids. The carbon source was 2% glucose or 2% galactose plus 0.02% glucose. For galactose induction of proteins, cells were first grown overnight in glucose containing medium and then diluted at 0.1 UA_600nm_ in galactose containing medium (with 0.02% glucose). For cell growth experiments, a culture was done overnight in glucose containing medium. The glucose cultures were diluted 50 times in galactose containing medium (with 0.02% glucose) and grown for 24 h. The galactose cultures were diluted at 0.1 UA_600nm_ in galactose containing medium (without glucose) and then cerulenin (Makor Chemicals Ltd) was added at 5 µg.mL^-1^ final concentration. All cultures were performed in conical flasks, containing 1/5 volume of medium, and incubated at 28°C in an orbital shaker at 200 rpm.

### Sequences and plasmid constructs

*A. thaliana* sequences used in this study were *AtOLE1* (At4g25140 gene and CAA44225 protein accession number), and *AtCLO1* (At4g26740 gene and AAL36241 protein accession number). To construct the pGAL-OLE1-GFP plasmid, the *AtOLE1* open reading frame of the pET20b-S3 plasmid [52] was amplified with the primers 5'-**gtacaaggatccatggcggatacagctagaggaac**-3' and 5'-**ataaatcgatagtagtgtgctggccacccac**-3'. The BamHI/ClaI PCR fragment obtained was then introduced into plasmid pNBT29 [50].

### Total protein extracts

Total proteins were extracted using the NaOH/trichloroacetic acid (TCA) lysis technique described by Volland et al. [53]. Cells (corresponding to 1.5 UA_600nm_ or 0.75 mg Dry Weight (DW)) were collected and resuspended in a final volume of 500 µL. 50 µL of 1.85M NaOH was added to the sample and cells were disrupted by vortexing and incubating on ice for 10 min. Proteins were precipitated for 10 min on ice by adding 50 µL of 50% TCA. The resulting protein pellet was resuspended in 50 µL of loading buffer containing 2 volumes of 2X sample buffer (100 mM Tris-HCl pH6.8, 4 mM EDTA, 4% SDS, 20% glycerol and bromophenol blue), 1 volume of Tris Base 1 M and 2% mercaptoethanol.

### Lipid droplet purification

Lipid droplets were separated by density gradients as previously described by Yu et al. [54]. Cells (corresponding to 400 AU_600nm_ or 160 mg DW) were harvested by centrifugation at 3000 x g for 10 min at 4°C. The pellet was washed with sterile water and resuspended in 3 mL of Fat Body Buffer (FBB, 10 mM Hepes, 10 mM KCl, 0.1 mM EDTA, 0.1 mM EGTA at pH7.5) supplemented with protease inhibitors (Complete cocktail, Roche Diagnostics). Cells were disrupted in a One Shot Cell Disrupter (Constant System LDT) at a maximum pressure of 2.7 kbars. The volume of cleared extract was adjusted to 2.7 mL with FBB, mixed with an equal volume of FBB including 1.08 M of sucrose, transferred to an 11 mL ultracentrifugation tube and overlaid sequentially with 1.8 mL each of 0.27 M and 0.135 M sucrose in FBB, and FBB only. After centrifugation at 150 000 x g for 90 min, 1.5 mL of floating lipid droplets were recovered from the top of the gradient. Proteins from purified lipid droplets were precipitated by adding 1.5 mL of 20% TCA and incubating on ice overnight. Proteins were collected by centrifugation at 10 000 x g for 30 min, resuspended in 400 µL of loading buffer. Proteins (1.4 µg) were separated by SDS-PAGE electrophoresis

### SDS-PAGE and immunoblots

Proteins were separated by SDS-PAGE using ready-to-use NuPage Novex 12% Bis-Tris gels and NuPAGE MES SDS running buffer (Invitrogen). Gels were stained with Coomassie blue (G-250) or with a silver staining kit (Invitrogen). For immunoblotting experiments, proteins were transferred to Immobilon-P PVDF membranes (Millipore) and detected with monoclonal antibodies raised against Act1 (Abcam), Dpm1(Abcam), Glycéraldéhyde-3-Phosphate Deshydrogenase (GAPDH) (Millipore), Gas1 (kind gift from H. Riezman), GFP (Roche Diagnostics), Pep1 (Abcam), Vam3 (Santa Cruz Biotechnology). Primary antibodies were detected using horseradish peroxidase-conjugated IgG secondary antibodies (anti mouse from Sigma-Aldrich, anti-rabbit from Pierce and anti-goat from SouthernBiotech) and revealed using SuperSignal West Dura Extended Duration Substrate (Perbio). Luminescence from peroxidase activity was recorded using the LAS-3000 imaging system and MultiGauge software from Fujifilm.

### Fatty acid quantification

Cells (corresponding to 50 UA_600nm_ or 20 mg DW) were collected by centrifugation, washed with water and freeze dried for 72 h. The pellet was disrupted by vortexing in presence of 0.45 mm glass beads and 2 mL of 2.5% (v/v) sulfuric acid in methanol. Heptadecanoic acid (Sigma-Aldrich) was added (100 µg for each sample) as internal standard for quantification. The samples were heated for 90 min at 80°C. Fatty acid methyl esters (FAME) were extracted by addition of 3 mL of water, 0.9 mL of hexane and vigorous shaking. Samples of the organic upper phase were separated by gas chromatography using a 7890A chromatograph (Agilent) with a Factor Four VF-23ms 30m*0.25 mm capillary column (Agilent). The carrier gas was helium at an inlet pressure of 1mL/min. The column temperature program started at 40°C during 1 min, ramping to 120°C at 40°C/min, holding 1 min at 120°C, ramping to 210°C at 3°C/min and holding 10 min at 210°C. Identification of FAME peaks was based upon retention times obtained for standards (Sigma-Aldrich). The quantification was made by flame ionization detection at 270°C. The total amount of fatty acids was calculated from the ratio between the sum of FAME peak areas and the heptadecanoic acid methyl ester peak area.

### Lipid analysis

Cells (corresponding to 200 UA_600nm_ or 80 mg DW) were processed according to Folch et al. [55]. Cells were harvested by centrifugation and washed with water. 5 mL of chloroform/methanol ratio 2/1 (v/v) were added to the pellet and the cells were disrupted by vortexing 5 min using glass beads (0.45 mm). After 1 h incubation with shaking, the extract was centrifuged 5 min at 500 x g and the supernatant was recovered in a new tube and mixed with 2.5 mL of 0.9% NaCl. The organic phase (lower phase) was collected after centrifugation at 500 x g for 5 min and washed three times with chloroform/methanol/water 3/48/47 (v/v/v) [56]. The organic solvents were evaporated under stream of N_2_ and lipids were resolubilized in 100 µL of chloroform/ methanol 2/1 (v/v). Lipids (20 µL) were separated by thin layer chromatography (TLC) on HPTLC silica coated aluminum plates (Merck) using successively two mobile phases, petroleum ether/diethyl ether/acetic acid 10/10/0.4 (v/v/v) and petroleum ether/diethyl ether 49/1 (v/v) until the solvent front reaches about 15 cm and 1 cm from the top of the plate, respectively [39,57]. Lipid classes were visualized by MnCl_2_ charring method. Silica plate was incubated 1 min in a solution containing 120 mL of methanol, 120 mL of water, 0.8 g of MnCl_2_ and 8 mL of sulfuric acid and then heated in an oven at 100°C until appearance of dark lipid spots. Lipid identification was based upon migration obtained for lipid standards (Sigma-Aldrich). Lipid staining was recorded using the LAS-3000 imaging system and MultiGauge software from Fujifilm.

### Electron microscopy

Cells (~ 12 UA_600nm_ or 5 mg DW) were fixed for 1 h at 28°C by adding 250 µL of 50% glutaraldehyde to 5mL of overnight galactose growing cells. After 5 min centrifugation at 3000 x g, cells were resuspended in 3% glutaraldehyde in Phosphate buffer and fixed overnight at 4°C. Then, cells were washed three times with 0.1 M sodium cacodylate pH7.4, three times with water, incubated in 1% KMnO_4_ for 2 h on ice, and again washed three times in water. Subsequently, cells were treated with 2% aqueous uranyl acetate for 1 h at 4°C and washed with water. Cells were gradually dehydrated in ethanol, and in propylene oxide, and embedded in Epon containing 2% dimethylaminoethanol (DMAE) (Delta Microscopies). Thin sections (70 nm) were cut, stained with lead citrate and examined in a Zeiss EM902 transmission electron microscope (Zeiss) at 80 kV. Micrographies were acquired using MegaView III CCD camera and analyzed with ITEM software (Eloise SARL).

### Fluorescence microscopy

GFP fluorescence was monitored in cells collected from cultures using the microscope without further treatment. For neutral lipid staining, lipid droplets were incubated for 10 min in HCS LipidTOX Neutral Red Lipid Stain (Molecular Probes) as recommended by the supplier. A Zeiss Axio Imager microscope with fluorescence and Nomarski optics and a Roper CoolSnap HQ2 camera coupled to a Zeiss AxioVision driver was used.

### Single cell yeast synchrotron microspectroscopy

Synchrotron FT-IR microspectroscopic analysis was performed at the SOLEIL synchrotron (Gif-sur-Yvette, France) using the SMIS beamline. Cells (1 mL of culture) were collected by centrifugation and washed once with water before analysis. Two microliter drops were dried under low vacuum on the Zinc Selenide (ZnSe) 4 mm diameter Attenuated Total Reflectance (ATR) hemispherical Internal Reflection Element (IRE) (ISP Optics Corp., Irvington, NY), providing a high spatial resolution (4x4 µm^2^). All spectra were recorded in reflexion mode on a Continuum XL microscope (Thermo, Fisher Scientific). The microscope comprises a motorized sample stage and a liquid nitrogen cooled mercury cadmium telluride (MCT-A) detector (50 µm element size). The microscope operates in confocal mode using a 32x infinity corrected Schwarzschild objective (NA=0.65) and a matching 32x condenser. All spectra were recorded using a dual mask aperture of 10 x 10 µm^2^. Individual spectra were saved in log(1/R) format at 8 cm^-1^ spectral resolution, with 128 co-added scans encompassing the mid-IR region from 4000 to 800 cm^-1^. We recorded 50 single cell IR spectra for each culture.

### Matlab pretreatment and RMieS correction (Matlab)

Spectra were imported into Matlab (MathWorks, v 7.1) software and Resonance Mie Scattering (RMieS) Extended Multiplicative Scatter Correction (EMSC) correction (RMieS_EMSC_v3) was applied [58–62]. Twenty algorithm iterations were used and the custom reference spectrum option chosen. The custom spectrum was selected from the set of raw data and chosen with no (or little) baseline distortion.

### Multivariate data analysis (PCA and PLSR)

Principal Component Analysis (PCA) is a powerful chemometric method to reveal variances or combination of variables among multivariate data. In PCA, the raw data matrix X (n samples times p wavenumbers) is centred and split into a sum of matrix product TP^-1^ and a residual matrix E. Where T is the score matrix and P^-1^ the corresponding loading matrix transposed. The score vectors (shown in score plot) represent the footprints of the objects projected down onto the principal components and the loadings vectors (shown in loading plot) can be viewed as the bridge between the variable space and principal component space. The characteristic absorption bands were revealed by studying the spectral patterns derived from the loadings plots. In Partial Least-Square Regression (PLSR) an additional design variables matrix is used as the Y-matrix. The Y-matrix is defined by using indicator variables 0 and 1 for the design factors (e.g. GFP, AtOle1-GFP and AtClo1-GFP). Thus, PLS uses the Y structure as a guiding hand in decomposing the X matrix. The FT-IR data of single cells were used for PCA and PLSR using the Nonlinear Iterative Projections Alternating Least Squares (NIPALS) algorithm (The Unscrambler 10.1, Camo, No). PCA was used to study the unsupervised variation in the data and PLSR to relate the spectral data to each yeast strain.

### Glycogen and trehalose quantification

The glycogen and trehalose content of each strain was determined as described in [63]. Cells (corresponding to 25 UA_600nm_ or 10 mg DW) were collected by centrifugation, washed with water and freeze dried for 72 h. Samples were then boiled for 4 h at 95°C in 250 µL of 0.25 M Na _2_CO_3_ then, 150 µL of 1 M acetic acid and 600 µL of 0.2 M sodium acetate were added to obtain a raw extract. For trehalose hydrolysis, trehalase (T8778, Sigma) was added at 0.073 u.mL^-1^ final concentration to 250 µL of the raw extract and the sample were incubated for 48 h at 37°C. For glycogen hydrolysis, α-amyloglucosidase (10115-1G-F, Sigma) was added at 0.10 u.mL^-1^ final concentration to 500 µL of the raw extract and the sample were incubated for 24 h at 57°C. After incubation, samples were centrifuged and free glucose was quantified using high-performance anion-exchange chromatography with pulse amperometric detection (HPAEC-PAD) in a carbohydrate analyzer system from Dionex, equipped with a ED40 electrochemical detector, a GP50 quaternary pump, a CarboPac PA1 column (4 x 250 mm) with a CarboPac PA1 guard column (4 x 50 mm), coupled to Chromeleon software. In order to elute the sample, an isocratic gradient of 50 mM NaOH with a flux rate of 1 mL.min-1 was applied for 20 min. The column was washed with 300 mM NaOH and equilibrated with 50 mM NaOH before each sample.

## Results

### AtClo1-GFP and AtOle1-GFP share structural properties and are the major proteins of lipid droplets when expressed in yeast

**Figure 1:**
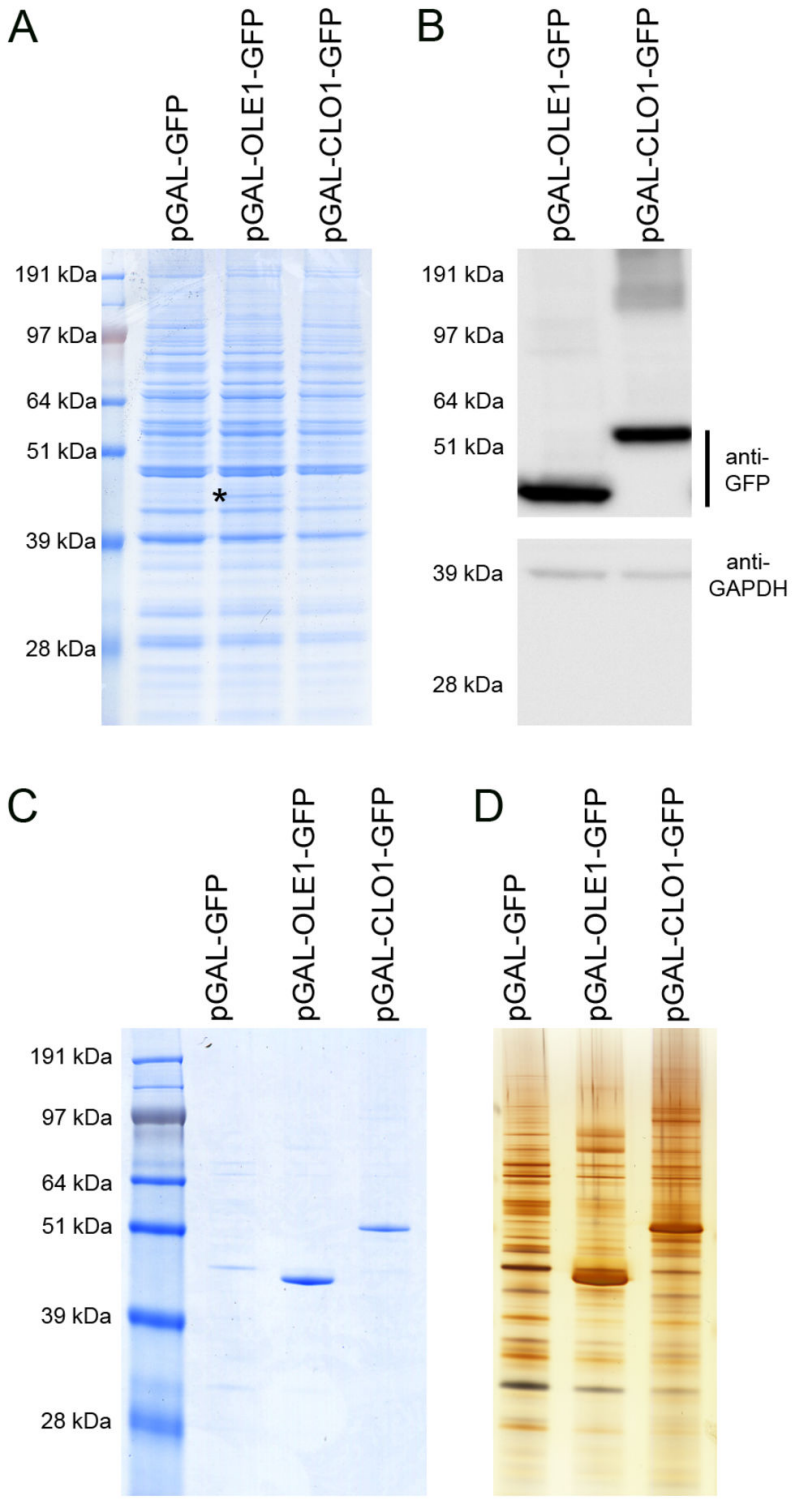
Expression of AtOle1-GFP and AtClo1-GFP in yeast. Total protein extracts from GFP, AtOle1-GFP and AtClo1-GFP expressing cells were analyzed using SDS-PAGE (A) and immunoblots (B). The presence of AtOle1-GFP (*) was visible on the Coomassie blue stained gel and the fusion proteins were also detected using an anti-GFP antibody. The anti-GAPDH antibody was used as a loading control. Association of the plant proteins with yeast lipid droplets was confirmed by SDS-PAGE analysis of the protein profile of purified lipid droplets. The proteins were revealed by Coomassie blue staining (C) or silver staining (D).

We previously expressed the *A. thaliana* minor lipid droplet integral protein, AtClo1, in *S. cerevisiae* which led to an increased lipid storage capacity in these cells. Targeting of the GFP-tagged protein to yeast lipid droplets increased the number and size of these organelles which resulted in a specific accumulation of neutral lipids (triacylglycerols and steryl esters) [32]. In the present study, we also used this GAL inducible heterologous expression system to perform a functional study of a GFP fusion of AtOle1, the major *A. thaliana* lipid droplet integral protein. We compared the effects of these two integral lipid droplet proteins, which share structural properties, on lipid droplet morphology and neutral lipid storage when expressed in yeast. We first used SDS-page of total protein extracts to verify whether the plant proteins were correctly expressed (presence, apparent molecular weight and amount) in yeast cells after 18 h of induction in galactose-containing medium. For AtOle1-GFP expressing cells, we observed a specific band at 46 kDa corresponding to the molecular weight of the fusion protein (Figure 1A). AtClo1-GFP was not detectable on Coomassie blue stained gels. However, immunoblot experiments on the same samples using antibodies against GFP confirmed expression of the two fusion proteins with specific signals at 46 kDa and 55 kDa for AtOle1-GFP and AtClo1-GFP, respectively (Figure 1B). We concluded that the two proteins were expressed at similar levels using anti-GAPDH as a loading control (Figure 1B).

**Figure 2:**
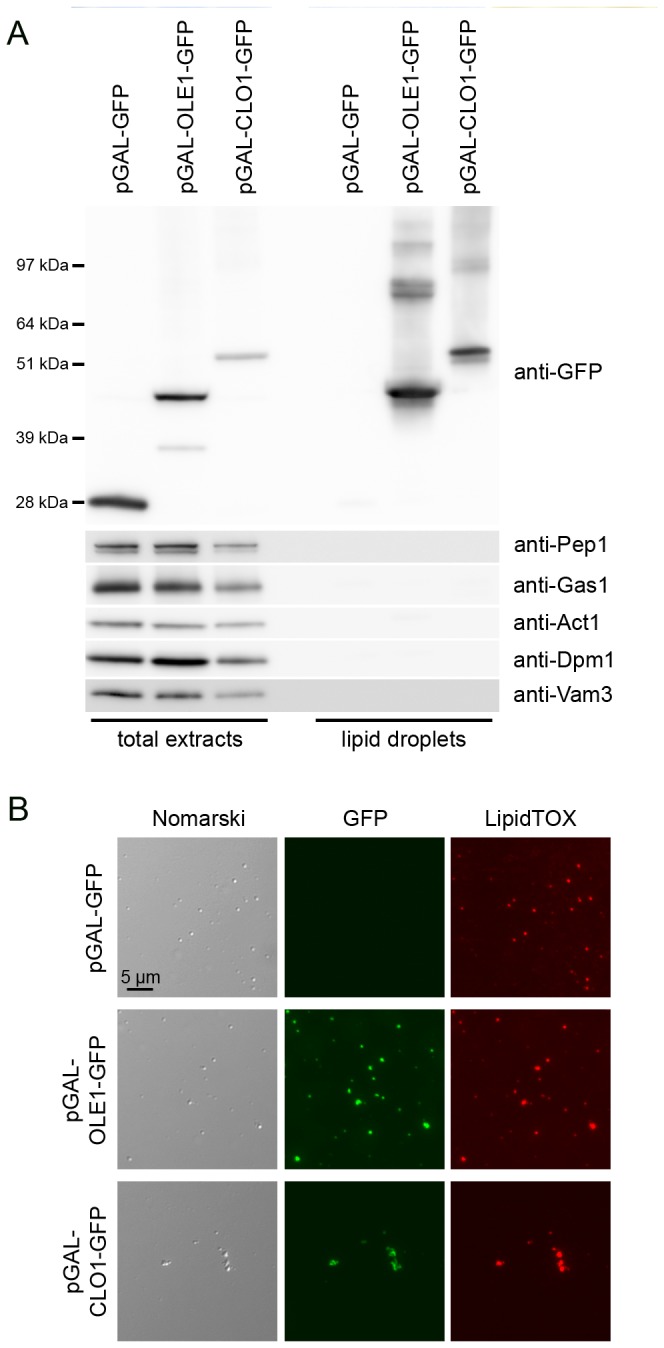
Quality control lipid droplets. Total protein extracts and purified lipid droplets from GFP, AtOle1-GFP and AtClo1-GFP expressing cells were analyzed using immunoblot (A) with antibodies specific for heterologous protein (anti-GFP), Golgi/endosome (anti-Pep1), plasma membrane (anti-Gas1), cytosol (anti-Act1), endoplasmic reticulum (anti-Dpm1) and vacuolar membrane (anti-Vam3). Purified Lipid droplets were observed with Nomarski optics (B, left panels) or by fluorescence with GFP (B, middle panels) or HCS LipidTOX Red Neutral Lipid Stain (B, right panels) filter sets.

We also evaluated the association of the expressed plant proteins with yeast lipid droplets using cell fractionation experiments. Lipid droplets from control cells and cells expressing AtOle1-GFP or AtClo1-GFP were purified by centrifugation on sucrose density gradients. A quality control of the fraction was performed using immunoblot experiments with antibodies against resident proteins of intracellular compartments (plasma membrane, endoplasmic reticulum, Golgi/endosome, vacuole and cytosol). Specific signals were observed for total protein extracts but nothing was detectable for lipid droplet fractions revealing no contamination with other intracellular compartments (Figure 2A). The purity of the fraction was confirmed using microscopy. We observed colocalization of HCS LipidTOX red signal with GFP signal revealing association of tagged-plant proteins with neutral lipids contained in lipid droplets (Figure 2B). Proteins in the floating fractions were separated by SDS-PAGE and stained (Coomassie and silver), revealing that the fusion proteins are the major proteins associated with lipid droplets (Figure 1C and D). We observed that oleosin and caleosin association with lipid droplets modifies the protein profile of the floating fraction with a partial or a total loss of some native proteins. Some high molecular weight forms, resistant to reductive agents, were also detected on immunoblots, probably corresponding to homodimers or heterodimers with endogenous lipids and/or proteins (Figure 2A). 

#### AtClo1-GFP and AtOle1-GFP expression have different effects on lipid droplet morphology and neutral lipid content in yeast

**Figure 3:**
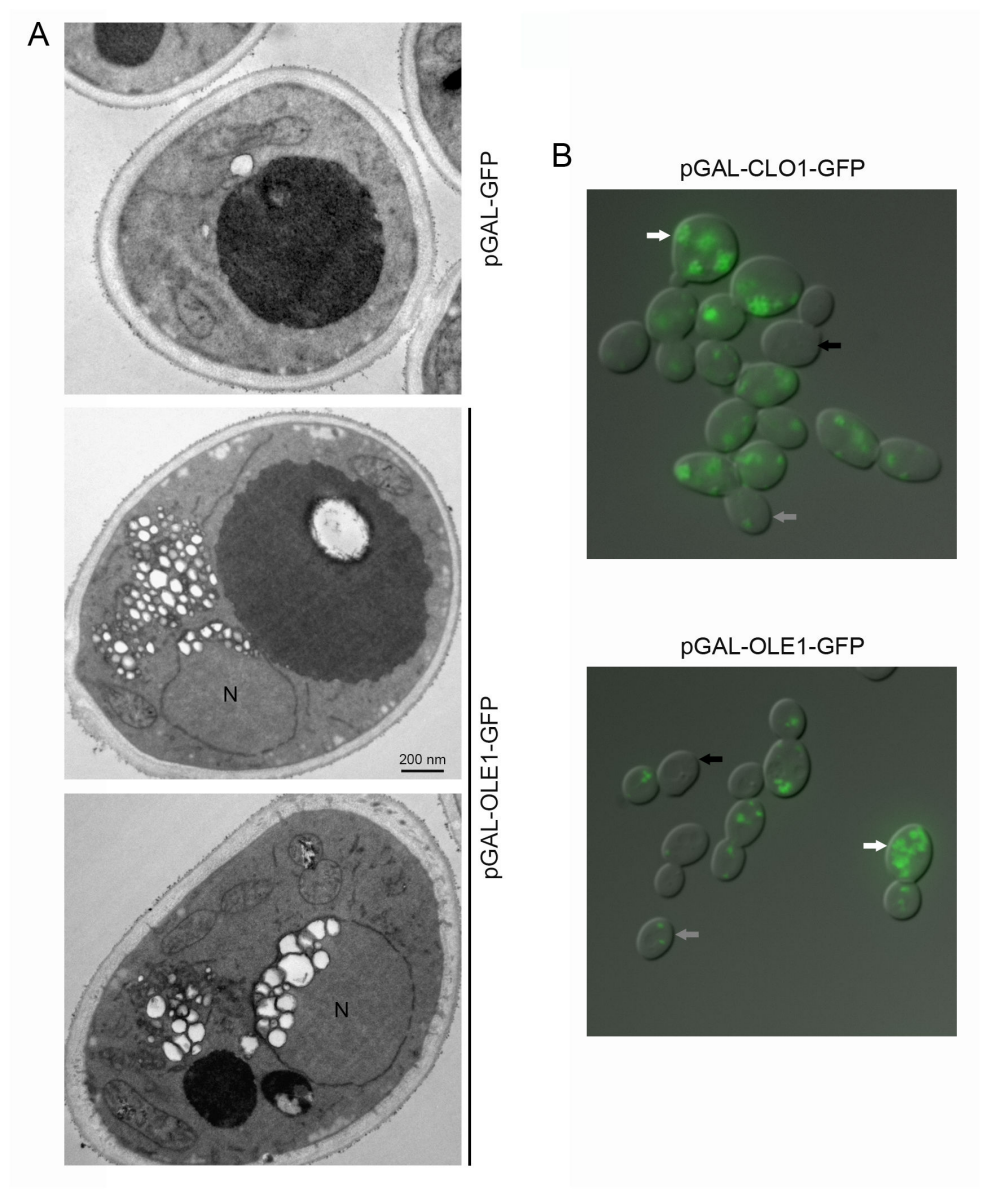
Observation of yeast cells expressing plant oleosin and caleosin. BY cells transformed with pGAL-GFP, pGAL-OLE1-GFP or pGAL-CLO1-GFP were cultured for 18 h in a galactose-containing medium. Cells were processed for electron microscopy (A). Lipid droplets appear as white round structures surrounded by a black membrane. N, nucleus. Cells were also observed for detection of GFP signal using fluorescence microscopy (B). White, grey and black arrows indicate cells with high, medium or no plant protein expression, respectively.

To investigate the effect of the expression of these plant proteins on lipid droplet morphology, we performed electron microscopy. Ultrastructural observation of thin sections revealed contrasting lipid droplet morphologies in plant protein expressing cells and in control cells. As previously reported [32], expression of AtCLo1-GFP in yeast cells induces an increase in the number and size of lipid droplets. In cells expressing AtOle1-GFP, we also observed significant lipid droplet proliferation but with specific accumulation under the nuclear envelope (Figure 3A). We also noticed cell-to-cell lipid droplet heterogeneity. We hypothesized that this heterogeneity was due to the inducible GAL promoter and its cell cycle dependant induction. Indeed, we observed that the plant proteins were not fully expressed in all cells using fluorescence microscopy (Figure 3B).

**Figure 4:**
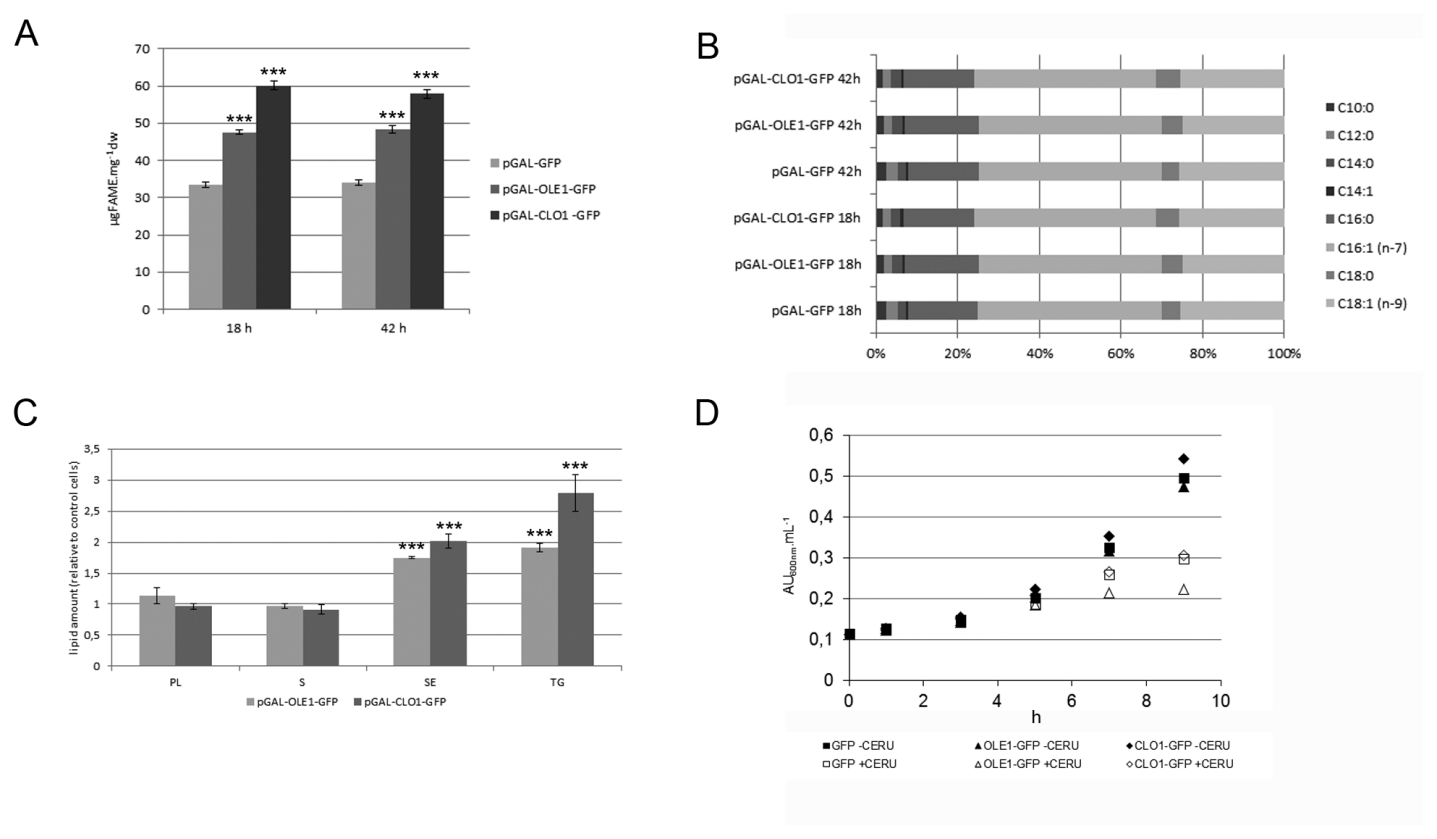
Comparative lipid analysis of yeast strains expressing plant oleosin and caleosin. Total fatty acid content of GFP, AtOle1-GFP and AtClo1-GFP expressing cells after 18 or 42 h induction in galactose-containing medium was evaluated by gas chromatography after FAME production of freeze-dried samples (A and B). Lipids were also extracted and spotted on silica plates for thin layer chromatography. Each class of lipid was quantified and data were plotted as the amount relative to that obtained for cells expressing GFP alone. Data were expressed as the mean ± SE of three experiments (C). *** Significant difference according to *t* test, P < 0.001. Growth curves of cells, transformed with pGAL-GFP (squares), pGAL-OLE1-GFP (triangles), pGAL-CLO1-GFP (diamonds) in the absence (filled symbols) or presence (open symbols) of 5 µg.mL ^-1^ cerulenin (inhibitor of fatty acid synthesis). The experiment was repeated twice (D).

We next investigated whether these lipid droplet modifications lead to variability in lipid accumulation. We analyzed the lipid content of GFP, AtClo1-GFP and AtOle1-GFP expressing cells after 18 or 42 h induction in galactose-containing medium. We first measured the total fatty acid content of cells using gas chromatography analysis. As reported previously [32], AtClo1-GFP increases lipid accumulation in yeast (58.5 ± 0.5 µg FAME.mg^-1^dry weight; n=3) compared to cells expressing GFP alone (33.5 ± 0.7 µg FAME.mg^-1^dry weight; n=3) after 18 h induction. For cells expressing AtOle1-GFP, the total fatty acid content also increased (47.6 ± 0.5 µg FAME.mg^-1^dry weight; n=3) but not to the same extent as for AtClo1-GFP-expressing cells (Figure 4A). The same fatty acid content was observed after 42 h induction in galactose-containing medium. No changes to the total fatty acid profile were observed (Figure 4B). To examine the nature of the lipids stored in these cells, we performed thin layer chromatography after Folch extraction of lipids from the same strains after 18 h. Quantification of the lipid classes revealed an accumulation of neutral lipids, triacylglycerols and steryl esters, specifically stored in lipid droplets. Altogether, our results showed that AtClo1-GFP expressing cells store fatty acids more efficiently than AtOle1-GFP expressing cells and these fatty acids are esterified with glycerol or sterol to form neutral lipids (Figure 4C). We also investigated the impact of plant protein expression on lipid and fatty acid metabolism. We followed cell growth in the presence of cerulenin, an inhibitor of fatty acid synthesis in yeast. It was published that in the presence of cerulenin, the fatty acids which are essential for initial growth cannot be obtained by *de novo* synthesis but only after hydrolysis of storage lipids (triacylglycerols and steryl esters) [64,65]. We observed that cerulenin had a negative impact on the growth of all the strains due to an absence of *de novo* fatty acid biosynthesis. We also observed that cells expressing AtOle1-GFP were more sensitive to cerulenin than the control and At-Clo1-GFP expressing cells (Figure 4D). With these observations we demonstrated that AtOle1-GFP and AtClo1-GFP do not have the same impact on fatty acid metabolism and probably on neutral lipid metabolism when expressed in yeast. Thus, these contrasting strains are powerful tools with which to study the metabolic changes induced by neutral lipid accumulation.

### Single cell synchrotron FT-IR microspectroscopy (sFT-IR) on contrasted strains revealed global metabolic changes induced by lipid accumulation

Due to the inducible GAL promoter (see below), we could hypothesize that the dynamics of the expression system could be extended with a minimum and a maximum of storage lipid overaccumulation per cell. To evaluate this plasticity it is essential to perform single cell measurement on global population. For this purpose, we used single cell synchrotron FT-IR microspectroscopy. This approach allowed us to perform statistical analysis (PCA and PLS) on the data collected. The score plot shows the samples (yeast single cells) plotted in a pertinent sub-space (principal components). Therefore a simple view, we are able to observe the variances of the data and facilitate interpretation of the meaning of the inter-object dispositions and sample heterogeneity.

**Figure 5:**
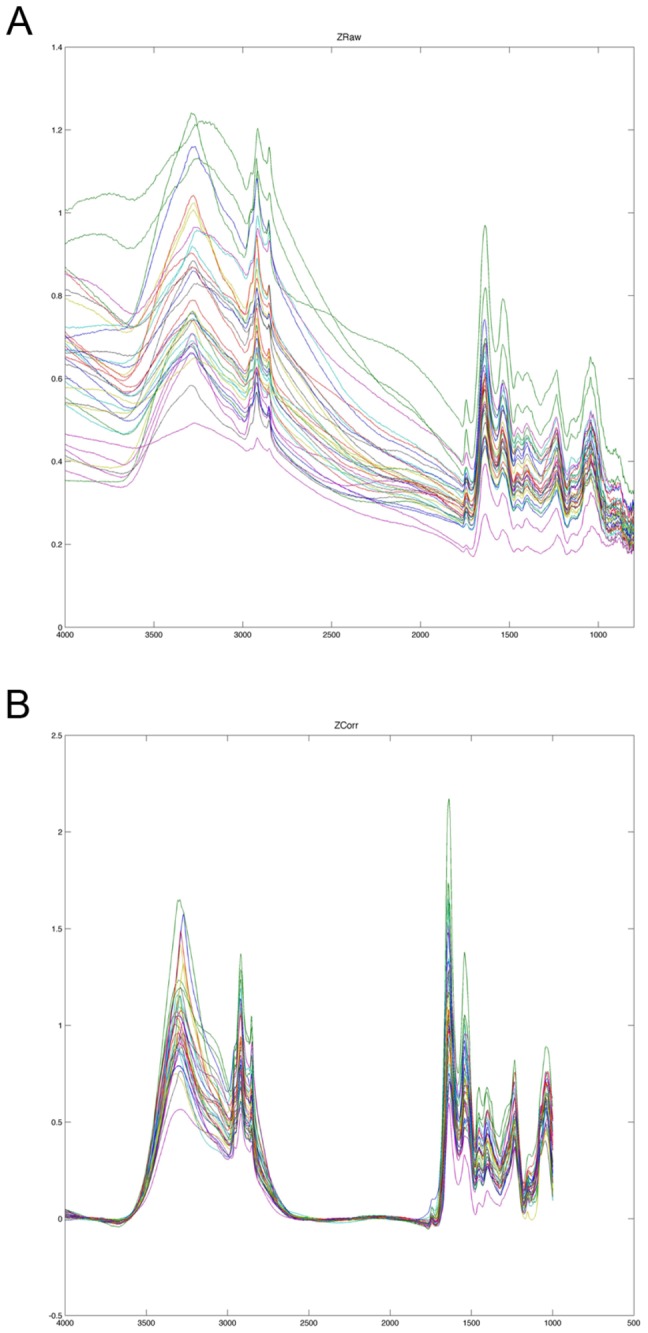
RMieS correction. Raw Spectra (A) from AtClo1-GFP expressing cells after 42 h of galactose induction were subjected to Resonance Mie Scattering (RMieS) EMSC correction to obtain pretreated spectra (B) which could be used for multivariate statistical analysis.

The sFT-IR spectrum of biological tissues has been extensively described within the scope of clinical diagnosis or fundamental biological studies. Thus, given well-characterized spectral peak positions and their biomolecule assignments are available in the literature [66–69]. We acquired spectra on single cells expressing GFP, AtOle1-GFP and AtClo1-GFP after 18 or 42 h of induction in galactose-containing medium. Due to light scattering, spectral artifacts were observed as derivative shape baseline on the recorded single cell spectrum (see in Figure 5A). Resonance Mie Scattering (RMieS) EMSC correction was therefore applied to all spectra (see experimental procedures). As shown in Figure 5B, the scattering effect was significantly reduced and the correction produced a consistent set of spectra.

**Figure 6:**
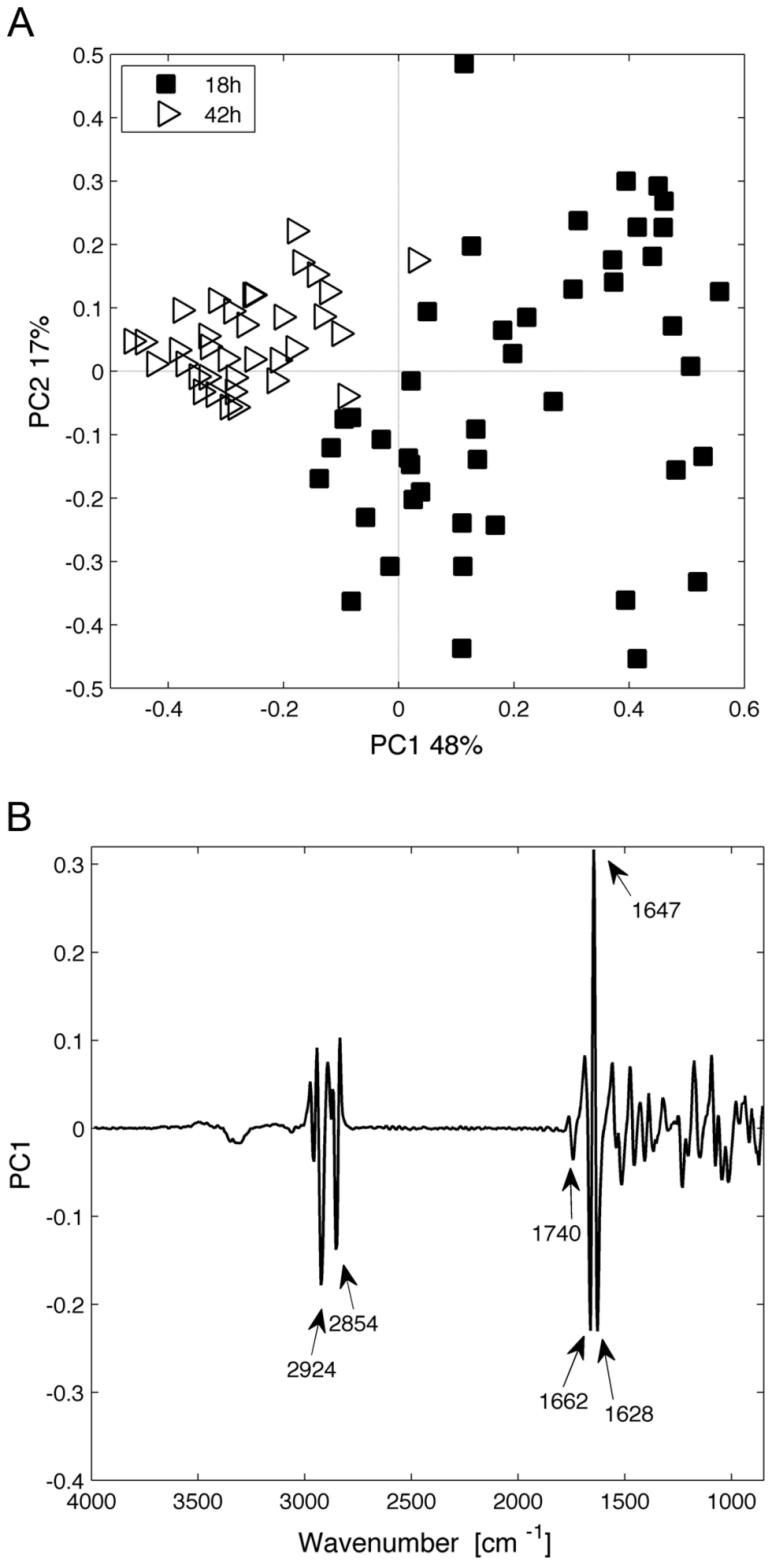
Multivariate statistical analysis (PCA) of spectra obtained for AtClo1-GFP expressing cells after 18 h or 42 h of galactose induction. (A) Score plot (principal component 1 (PC1) versus principal component 2 (PC2)). Each spectrum is represented by a label and plotted in a pertinent principal component sub-space. (B) The corresponding loading plot of the PC1 axis is presented. The loading plot links the variable space and principal component sub-space (PC1).

Since we did not observe changes in total fatty acid content between 18 h and 42 h of culture using gas chromatography, we investigated whether sFT-IR microspectroscopy could reveal some differences between these two culture conditions. The results obtained for AtClo1-GFP expressing cells after 18 or 42 h of galactose induction were compared. PCA analysis on single cell recorded data revealed a clear separation of the two populations along the PC1 axis with a stronger heterogeneity for the population induced for 18 h (Figure 6A). The corresponding PC1 loading (Figure 6B) showed high intensities at 1740, 2854 and 2924 cm^-1^. We attributed these C=O and C–H stretching infrared band variations (Table 1) to a higher fatty acid content or a modification in fatty acid composition in the 42 h population. Thus, we selected this population for the following studies. High loading values were also observed at 1647, 1662 and 1628 cm^-1^. These could be attributed to modifications in protein secondary structure.

**Table 1:** Spectral interpretation after multivariate statistical analysis on cell populations.

**Wavenumber (cm^-1^)**	**Assignment** [66-69]
891	C-C, C–O deoxyribose, fatty acid, saccharide
1008	Torsion C=C
1030	Glycogen
1072	Glycogen, phosphate
1100	Carbohydrates, phosphate
1151	Glycogen
1230	Amide III
1250	Amide III
1404	Symmetric CH_3_ (proteins)
1470	CH_2_ bending of the methylene chains in lipids
1575	C=N adenine
1620	Amide I
1628	Amide I
1639	Amide I
1647	Amide I
1655	Amide I
1662	Amide I
1740	Ester (lipids)
2854-2850	Symmetric CH_2_ (lipids)
2924-2920	Asymmetric CH_2_ (lipids)
2962-2970	Asymmetric CH_3_ (lipids)

**Figure 7:**
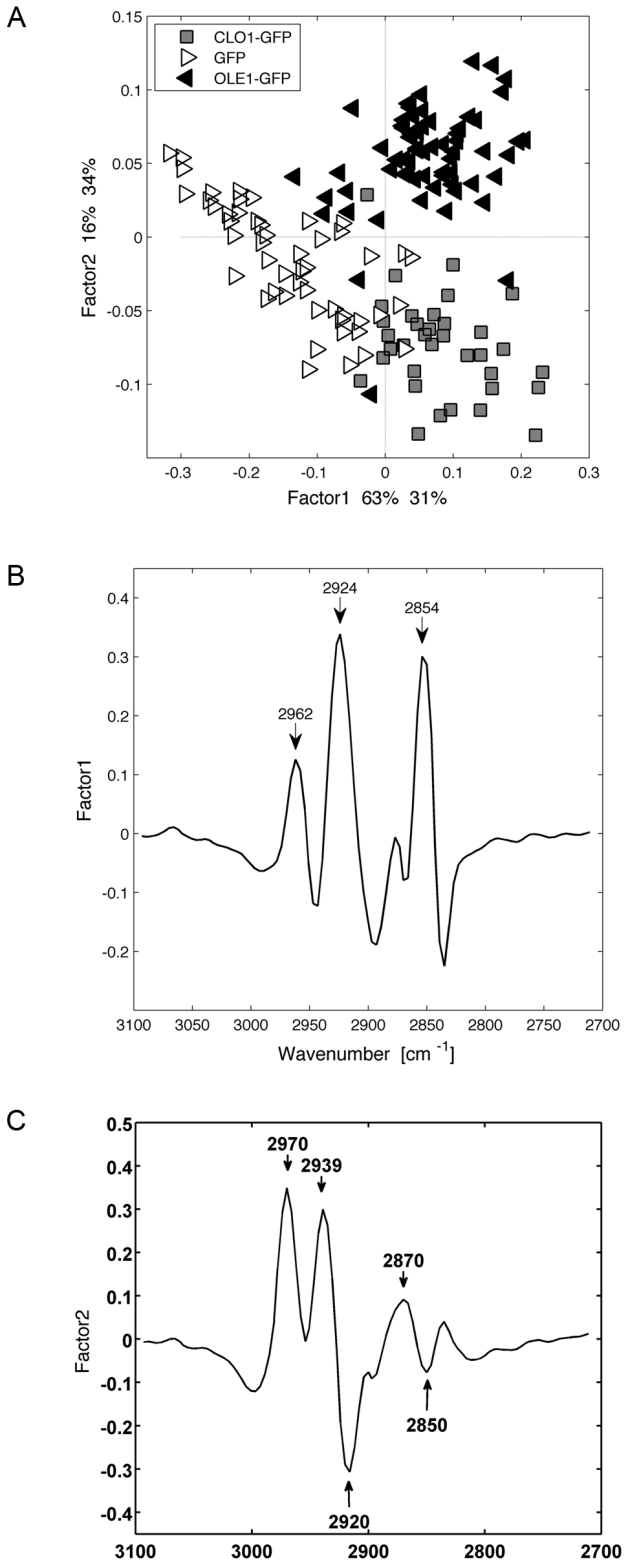
Multivariate statistical analysis (PLS) of spectra obtained for GFP, AtOle1-GFP and AtClo1-GFP expressing cells after 42 h of galactose induction. Score plot (A) of the factor 1 and factor 2 axes and the corresponding loading plots of the factor 1 (B) and factor 2 axes (C) are presented.

We then analyzed single cells expressing GFP, AtClo1-GFP or AtOle1-GFP after 42 h of galactose induction. We focused our attention on characteristic vibrations corresponding to lipid components, i.e. between 2840 cm-1 and 3000 cm^-1^ (C–H, CH_2_ and CH_3_ of fatty acids, Table 1). We detected significant differences in lipid storage between control and modified strains and confirmed a clear correlation between plant protein expression and overaccumulation of lipids in the cell populations (Factor 1, Figure 7B). Along the Factor 2 axis in the PLS (Figure 7C), shifts in C–H stretching vibration region (2920, 2850 cm^-1^) separated the two strains. These shifts could be attributed to change in lipids composition that may have masked the difference in lipid content detected using gas chromatography.

**Figure 8:**
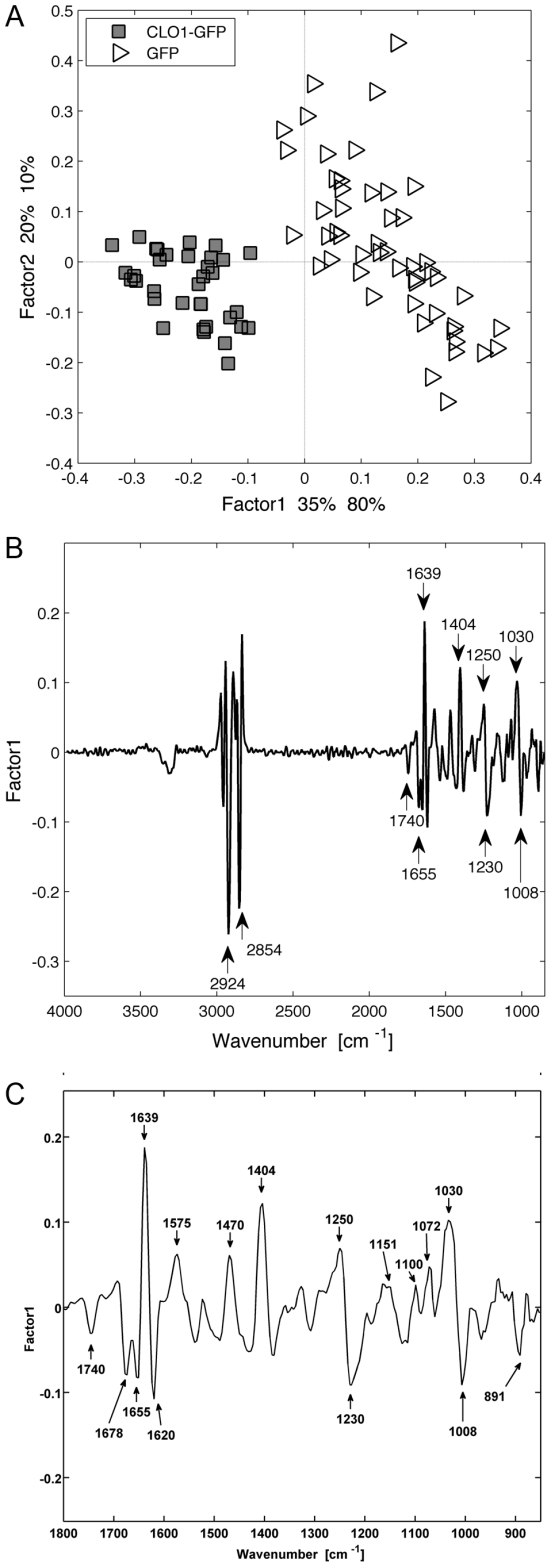
Multivariate statistical analysis (PLS) of spectra obtained for GFP and AtClo1-GFP expressing cells after 42 h of galactose induction. Score plot (A) of the factor 1 and factor 2 axes and the corresponding loading plot of the factor 1 axis (total range in (B) and zoom between 850 and 1800 cm^-1^ in (C)) are presented.

Following these observations, we decided to use the set of data collected for the GFP expressing control cells after 42 h and for AtClo1-GFP expressing cells, as these had the higher fatty acid and neutral lipid content as revealed in the chromatography experiments. PLS analysis on the full spectral range of these two populations showed a correlation between lipid specific absorption band variations (Figure 8 and Table 1) and additional band variations. In particular, we found a direct correlation between lipid accumulation and the 1008 cm^-1^ band attributed to C=C, potentially due to an increased unsaturated fatty acid content. We detected some shifts in the regions corresponding to Amide stretching bands suggesting some protein structure modifications (Figure 8 and Table 1). We also noticed an inverse correlation between some of the lipid bands and the region between 1020 and 1150 cm^-1^ assigned to carbohydrate stretching and more specifically to glycogen vibrations (1151, 1100, 1072 and 1030 cm^-1^) (Figure 8 and Table 1).

### Neutral lipid overaccumulation in AtClo1-GFP and AtOle1-GFP expressing cells is correlated with a decrease in glycogen and trehalose storage

**Figure 9:**
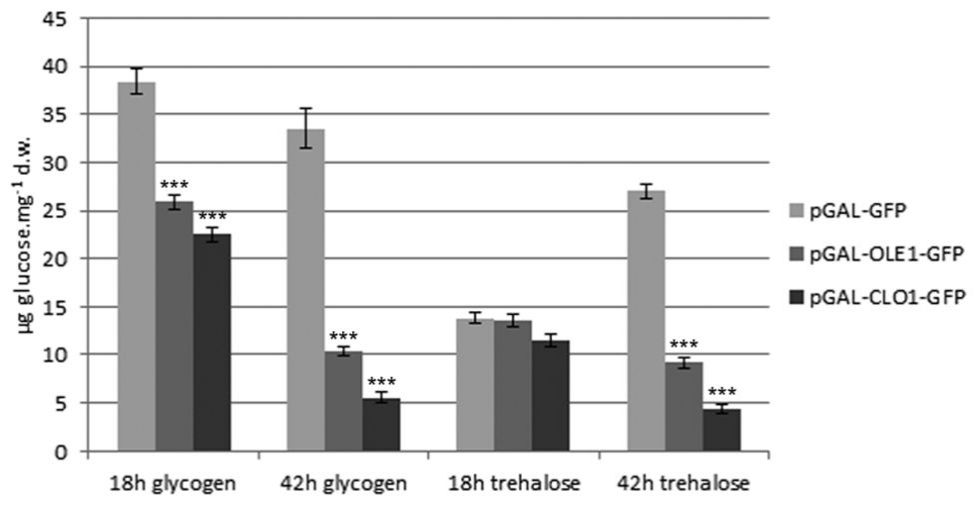
Glycogen and trehalose analysis of yeast cells expressing plant oleosin and caleosin. The storage carbohydrate, i.e. glycogen and trehalose, content of control, GFP, AtClo1-GFP and AtOle1-GFP expressing cells after 18 or 42 h induction in galactose-containing medium was evaluated using high-performance anion-exchange chromatography after specific enzymatic hydrolysis of freeze-dried samples. Data were expressed as the mean ± SE of three experiments. *** Significant difference according to *t* test, P < 0.001.

To confirm the decrease in glycogen observed in our strains using FT-IR, we quantified the storage carbohydrates, glycogen and trehalose, in GFP, AtOle1-GFP or AtClo1-GFP expressing cells after 18 and 42 h induction in galactose-containing medium (Figure 9). Glycogen content decreased for all strains between 18 and 42 h. For trehalose, the control and plant protein expressing strains behaved quite differently. In control cells, trehalose increased between 18 and 42 h, whereas a decrease was observed for cells expressing the plant proteins. Nevertheless, a decrease in the content of both storage carbohydrates at both culture times was observed for the plant protein expressing cells compared to the control. There also appeared to be a strong correlation between neutral lipid accumulation and the decrease in storage carbohydrates since the phenotype was more pronounced in AtClo1-GFP expressing cells which have the highest lipid content (Figure 9).

## Discussion

In this study, we confirmed previous observations that a GFP-tagged version of the caleosin AtClo1, a minor protein of *A. thaliana* seed lipid droplets induces neutral lipid accumulation when expressed in *S. cerevisiae*. A 74% increase in total fatty acid content was measured in these cells. We also demonstrated that a GFP-tagged version of the oleosin AtOle1, the major protein of *A. thaliana* seed lipid droplets, promotes triacylglycerol and steryl ester storage. Gas chromatography revealed that the storage efficiency was not as good as for AtClo1-GFP, with only a 42% increase in total fatty acid content, even if AtOle1-GFP was strongly associated with purified lipid droplets. Recently, a peanut oleosin isoform was reported to have both monoacylglycerol acyltransferase and phospholipase A2 activity [31]. The authors observed an increase in diacyglycerols and triacylglycerols and a decrease in phospholipids when the protein was expressed in yeast. Our results, using the *A. thaliana* oleosin, are partially in agreement with their observations. However, we did not measure an effect on phospholipid content using thin layer chromatography. Caleosins have been previously reported to have peroxygenase activity and signaling roles rather than lipid biosynthesis or degradation activity [70,71]. However, these interfacial proteins from seed lipid droplets share original structure for integral membrane proteins: two hydrophilic C-and N-terminal regions interrupted by a long (around 70 non polar residues) central hydrophobic region. Previous *in vitro* experiments on oleosin and caleosin revealed the importance of the hydrophobic central core, and a proline knot motif, for protein folding in the ER and subsequent targeting to lipid droplets [15,17]. No homologues or functionally equivalent molecules were identified in *S. cerevisiae* lipid droplets. Nevertheless, the yeast cellular machinery could recognize intrinsic information carried by the central part of the protein, most likely in the hydrophobic region, and this information was sufficient to properly target oleosin and caleosin to lipid droplets. We also observed that the insertion of plant protein in yeast lipid droplet modify the protein profile of this intracellular compartment. We suppose that plant proteins, due to their hydrophobic central domains, are tightly associated with lipid droplet. This strong anchoring of caleosin and oleosin could modify the surface of the organelle and impair the association of endogenous yeast proteins, depending on the nature of the interaction with lipid droplet (integral membrane protein, surface protein, protein/protein interaction).

Several authors hypothesized that oleosins are derived from caleosin because caleosin has been found in lipid droplets from more primitive species, such as algae and fungi which do not contain oleosins. Also caleosin is as efficient as oleosin in stabilizing lipid droplets in these organisms [72]. Our results obtained on yeast expressing AtOle1-GFP or AtClo1-GFP support this hypothesis. Indeed, correct organization of either caleosin or oleosin in the membrane produced a specialized interface with lipid droplet identity that led to an overaccumulation of neutral lipids. These results confirm that integral lipid droplet proteins are relevant biotechnological targets for improving neutral lipid storage in cells.

A combination of synchrotron radiation and the ZnSe hemispherical IRE was used to obtain single-cell FTIR spectra on our two strains with contrasting neutral lipid storage capacities. Thus, based on their chemical signature, the two strains have been clearly separated and their heterogeneity revealed. Due to the round shape, small size of yeast cells (5 µm diameter) and the wavelength range of mid-infrared (from 1 to 10 µm), we observed strong scattering and distortion of spectrum. However, we successfully applied the Resonant Mie Scattering (RMies) tool developed by Bassan et al. (see experimental procedures) for spectrum correction and made a comparative study of our biological samples.

Matlab pretreatment of spectra followed by PCA and PLS analysis pinpointed clear biochemical differences in the strains. Thus, single cell FT-IR is an effective approach for a thorough overall exploration of cell phenotypes which can then be used to target a specific metabolic pathway for further in-depth metabolomics or biochemical approaches. The analysis of our three contrasting yeast strains also revealed shift in the amide bands that indicates a change in protein secondary structure probably due to high expression of the plant proteins. Surprisingly, we were not able to highlight the difference in fatty acid content between the AtOle1-GFP and AtClo1-GFP expressing cells using sFT-IR. This was probably due to the shift in C–H stretching vibrations bands observed between AtOle1-GFP and AtClo1-GFP spectra. In contrast, using multivariate statistical analysis on sFT-IR spectra, we were able to discriminate AtClo1-GFP populations. It was not the case using gas chromatography. Therefore, the two approaches are not sensitive to the same metabolic modifications and seem to be complementary.

An inverse correlation was observed between lipid accumulation and storage carbohydrate content. These observations were confirmed using glycogen and trehalose assays. These results demonstrate that neutral lipid and storage carbohydrate fluxes are tightly connected and co-regulated. This coordination between cellular energy reserves is certainly dependent on the Snf1/AMP activated kinase as this enzyme controls on one hand, the synthesis and metabolism of lipids and on the other hand, glycogen storage and mobilization via the phosphorylation of enzymes from each pathway (Acetyl-CoA carboxylase, glycogen synthase, etc) [73,74]. It is also well established that the vacuole and autophagy play a key role in glycogen pool maintenance and in neutral lipid turnover [75–77]. Therefore, carbohydrate metabolic pathways and regulators appear to be relevant biotechnological targets for improving neutral lipid storage in yeasts.
